# Changes in detection of birth defects and perinatal mortality after introduction of prenatal ultrasound screening in the Kola Peninsula (North-West Russia): combination of two birth registries

**DOI:** 10.1186/s12884-015-0747-1

**Published:** 2015-11-23

**Authors:** Vitaly A. Postoev, Andrej M. Grjibovski, Evert Nieboer, Jon Øyvind Odland

**Affiliations:** Department of Community Medicine, UiT- The Arctic University of Norway, Tromsø, Norway; International School of Public Health, Northern State Medical University, 163061 Troickij av 51 NSMU, ISPHA, office 2519, Arkhangelsk, Russia; Department of International Public Health, Norwegian Institute of Public Health, Oslo, Norway; Department of Preventive Medicine, International Kazakh-Turkish University, Turkestan, Kazakhstan; North-Eastern Federal University, Yakutsk, Russia; Department of Biochemistry and Biomedical Sciences, McMaster University, Hamilton, ON Canada; Faculty of Health Sciences, School of Health Systems and Public Health, University of Pretoria, Pretoria, South Africa

**Keywords:** Prenatal diagnostics, Screening, Ultrasound diagnostic, Birth defects, Murmansk County Birth Registry, Perinatal mortality, Prenatal detection rate, Russia

## Abstract

**Background:**

Prenatal diagnostics ultrasound was established in Russia in 2000 as a routine method of screening for birth defects. The aims of the current study were twofold: to assess changes in birth defects prevalence at birth and perinatal mortality after ultrasound screening was implemented and to estimate prenatal detection rates for congenital malformations in the city of Monchegorsk (Murmansk County, North-West Russia).

**Methods:**

The Murmansk County Birth Registry and the Kola Birth Registry were the primary sources of information, and include 30 448 pregnancy outcomes in Monchegorsk for the period 1973–2011. Data from these registries were supplemented with information derived from hospital records about pregnancy terminations for 2000–2007.

**Results:**

The total number of newborns with any kind of birth defects in Monchegorsk during 1973–2011 was 1099, of whom 816 were born in the 1973–2000 period. The prevalence of defects at birth increased from 34.2/1000 (95 % CI = 31.9-36.5) to 42.8/1000 newborns (95 % CI = 38.0-47.7) after prenatal ultrasound screening was formally implemented. We observed significant decreases (*p* < 0.05) in the birth prevalence of congenital malformations of the circulatory system, the musculoskeletal system (including deformations), and other (excluding multiple); those of the urinary system increased from 0.9/1000 to 17.1/1000 (*p* < 0.0001). The perinatal mortality among newborns with any kind of malformation decreased from 106.6 per 1000 newborns with birth defects (95 % CI = 84.3-129.1) to 21.2 (95 % CI = 4.3-38.1). Mothers who had undergone at least one ultrasound examination during pregnancy (*n* = 9883) had a decreased risk of having a newborn die during the perinatal period [adjusted OR = 0.49 (95 % CI = 0.27-0.89)]. The overall prenatal detection rate was 34.9 % with the highest for malformations of the nervous system.

**Conclusion:**

Improved detection of severe malformations with subsequent pregnancy termination was likely the main contributor to the observed decrease in perinatal mortality in Murmansk County, Russia.

## Background

Prenatal diagnosis is the identification of birth defects prior to delivery and constitutes a routine practice in most of European countries [1]. Prenatal screening (PS) is now recognized as the key issue in the early detection and correction of birth defects (BD) [2]. The first assessments of impacts of PS on perinatal mortality were presented in the early 1990s. They were based on randomized control studies and considered controversial. While a study in Helsinki showed a reduction in perinatal mortality [3], this outcome was not supported by the findings of the multicenter Radius trial in the USA [4].

The effectiveness of PS can be estimated by the prenatal detection rate, which represents the proportion of BD recognized before delivery. It depends on the type of defect, quality of equipment and experience of the operator. According to the European Surveillance of Congenital Anomalies (EUROCAT) data, 64 % of BD could be diagnosed before birth although it varies widely, from 94 % for anencephaly to 87 % for omphalocele, and as low as 27 % for the transposition of great vessels [5]. For chromosomal anomalies, the reported average ultrasound detection rates ranged widely: 5.7 % (Klinefelter syndrome) to 78.6 % (triploidy); for Down syndrome it was 26.4 % [6], and 19.9 % for cardiovascular malformations (11 % for isolated cases and 40 % for multiple malformations) [7].

The PS of malformations has an influence on the stillbirth rate, as they lead to terminations of pregnancies when severe defects are diagnosed. Surgical correction of defects during the first days of life can also reduce neonatal mortality [8].

In Russia, prenatal ultrasound examination was established in the early 1990s, and became part of a screening program offered to all pregnant women in compliance with a national law promulgated in 2000. It is free of charge and is offered by obstetricians to every pregnant woman. Refusal must be put in writing. The Russian Ministry of Health stipulates a two-stage approach to PS [9]. Initially, PS applies to all pregnant women and is carried out in obstetrics out-patient departments. It includes ultrasonography between weeks 10–14, 20–24 and 30–32 of gestation. During the second trimester, biochemical tests of blood alpha-fetoprotein and chorionic gonadotropin are administered. The second stage involves regional medical-genetic consultations and measures directed at establishing the exact diagnosis and prognosis of the fetus. Different approaches could be adopted at this stage involving non-invasive (ultrasound, cardiotocography, Doppler mapping) and/or invasive (amniocentesis, chordocentesis, placentocentesis, and chorion aspiration) procedures as required. If a diagnosis of BD is confirmed, an Advisory board (Perinatal Consilium) suggests a future management plan, or advises pregnancy termination in case of incurable BD. Nevertheless, the final decision about how to proceed is made by the woman. According to Russian law [10], termination of pregnancy in case of BD is possible at any gestational age, but a woman’s decision to do so late in the pregnancy needs approval by the Perinatal Consilium. In case of chromosomal anomalies, termination is possible until and including week 22, later decision needs approval.

Ten years after the establishment of PS in Russia, it is pertinent to assess its efficiency in the detection of BD and impact on pregnancy continuations. However, the available information is limited. In 2001–2003, researchers from the Komi republic (Northwestern District) estimated a prenatal detection rate based on more than 29 thousands pregnancies, and reported that it was the highest (74.5 %) for neural tube defects and lowest (4.7 %) for cardiovascular malformations; for Down syndrome it was 33.3 % [11]. Another study in Samara (Volga District) noted that the occurrence of abortion in case of lethal BD increased from 8.75 % in 2000–2001 to 24.20 % in 2004–2007 [12].

A countywide population-based birth registry established in 2005 for Murmansk County provides a unique opportunity to study BD epidemiology at the population level [13]. Since living in an industrialized region of the Northwest Russian Arctic might influence reproductive health and pregnancy outcomes, we selected the city of Monchegorsk located in the Kola Peninsula for analysis. We previously reported a steady increase during 2003–2011 in the birth prevalence of BD for this community, with significant growth in the birth prevalence of defects of the nervous system and urinary malformations. It was suspected that one of the underlying reasons for the increase was the establishment of PS [14]. The objectives of the present study are: (i) determine the influence of PS on the prevalence at birth of BD and perinatal mortality, and (ii) estimate the prenatal detection rates for all groups of BD.

## Methods

### Study design and sources of information

Our study population includes all newborns delivered in Monchegorsk during the period 1973–2011 and registered either in the Kola Birth Registry (KBR) or in the Murmansk County Birth Registry (MCBR).

The KBR was established in 1998 by a retrospective retrieval from hospital delivery department records of information about all births from week 28 of gestation on that occurred in Monchegorsk as of March 1973. Registration continued prospectively until the end of 2005. Details of its construction, description, suitability for epidemiological studies and selected applications are available [13, 15, 16]. The MCBR was established in 2005, and prospectively registers pregnancy outcomes from 22 weeks of gestation on as January 1, 2006 [17]. The technicalities of merging the two registries were presented previously, as well as the Monchegorsk perinatal statistics [14].

Routine prenatal ultrasound examinations began in Monchegorsk in 1994 [16], although some earlier cases were recorded in the KBR. The latter were associated with severe pregnancy complications that were usually performed after 22 weeks of gestation, and consequently cannot be considered as constituting prenatal diagnostics. Until 2000, ultrasound screening was performed during the 19^th^ week of pregnancy in cases with medical indications [16]. According to the KBR data, 4718 women (70.6 %) had ultrasound examinations during pregnancy in the period 1994–2000, but this figure varied from 47.5 % in 1994 to 94.8 % in 2000. From 2001 on, the local hospital abided by the mentioned National law and conducted three examinations during pregnancy [9]. The information about ultrasound examination was recorded in the registry, and included gestational age at examination as well as the pathological findings. In the MCBR, gestational age at the first examination is registered, and the pathological findings from all examinations are indicated. However, the MCBR does not contain information about biochemical screening, as it only became part of clinical practice in the early 2000s; it had relatively low coverage during the implementation period.

In order to obtain a precise estimate of the prenatal detection rate, the data from the registries were supplemented by information from the Monchegorsk Municipal Hospital for the years 2000–2007 concerning terminated pregnancies linked to BD at gestational ages less than 28 weeks. It included the results of PS (including diagnosis), gestational age at diagnosis and termination. Such data were not available for 2008–2011 and, consequently, prenatal detection rates were limited to the 2000–2007 period.

### Data analysis

To compare changes before and after PS implementation, the prevalence of BD at birth were calculated for the periods 1973–2000 and 2001–2011 using data from the two registries. We also assessed the birth prevalences of BD for which reporting is mandatory in Russia. They include the 22 most severe forms of BD as described in detail in our previous publication [14]. At this stage, all newborns were included for whom there was information about BD and their status at birth and during the first week of life (*n* = 30 448).

We stratified perinatal mortality for all newborns (with and without BD) into three time-periods: 1973–2000, 1990–2000, and 2001–2011. It was calculated as the sum of stillbirths and deaths during the first week of life per total births. Differences in birth prevalences and mortality rates within these time-periods were analyzed by the chi-squared test. We considered the time span between 1990 and 2000 to have been influenced by slow technical progress and changing registration methods.

The effect of PS on perinatal mortality was estimated by logistic regression. Altogether, 10 317 newborns out of 10 502 born in the years for whom ultrasound technology was available (1994 or later) were included in the analysis. Of these newborns, 185 (1.8 %) had missing information; gestational age was missing for 103 (1.0 %), 76 (0.7 %) had no information about PS, and both fields were missing in six (0.1 %). Perinatal death was included as a dependent variable, while ultrasound screening was a binary variable with positive values if at least one ultrasound examination had occurred. Gestational age, year of birth, maternal age and previous history of perinatal deaths were included in the model as independent variables (all were tested for multi-collinearity).

We estimated prenatal detection rates for all groups of BD according to The International Statistical Classification of Diseases and Related Health Problems 10th revision (ICD-10) for 2000–2007, and compared the PS data with perinatal diagnoses. The information available from the statistical department of the local hospital about pregnancy terminations due to fetal anomaly (TOPFA) were included in the analysis at this stage. Rates were calculated as the total number of newborns with BD diagnosed during ultrasound screening *divided by* the total number of newborns with BD. The newborns were included in the numerator only when the diagnosis at screening, at birth, and during the first week of life matched. The effect of TOPFA on perinatal mortality and on the stillborn rate were estimated by computing rates that combined registry data with the pertinent hospital information.

All statistical analyses were performed using SPSS 21.0.

### Ethical considerations

No personal maternal information such as first name, surname or address is recorded in the registries, so written consent could not be obtained from the mothers. The KBR was established retrospectively [13, 15, 16], with approval from the Murmansk County Health Authority (MCHA). In case of the MCBR, the MCHA passed legislation making birth registration and collection of pertinent medical data from hospital records mandatory. Nevertheless, each woman was informed at the first antenatal visit about the inclusion of such information in the registry database [17]. The creation of both registries and its protocols were approved by the Murmansk County Committee for Research Ethics (Murmansk, Russia) and the Regional Committee for Medical and Health Research Ethics (Tromsø, Norway). The latter and the Committee for Research Ethics at the Northern State Medical University (Arkhangelsk, Russia) approved the current study.

## Results

### Prevalence of birth defects at birth before and after the establishment of PS

There were 1099 newborns with any kind of BD during 1973–2011 in Monchegorsk; 816 of them were born in the pre-screening period (1973–2000) and 283 were born after its formal implementation (Table 1). The total prevalence at birth increased 24 % after PS was formally implemented (42.8 per 1000 newborns *versus* 34.2 per 1000 newborns). By contrast, the corresponding prevalence of the most severe defects (reporting of which is mandatory) did not change (Table 1).

**Table 1:** The total prevalence of malformations at birth before and after prenatal screening was formally instituted

Number or Prevalence	1973-2000	2001-2011	p-value^1^
Number of newborns included in the analysis	23 822	6626	
Number of newborns with BD	816	283	
Prevalence of BD at birth (per 1000 newborns)^2^	34.2 (31.9-36.5)^2^	42.8 (38.0-47.7)	0.001
Number of newborns with forms of BD requiring mandatory reporting	175	48	
Prevalence at birth of BD forms requiring mandatory reporting (per 1000 newborns)^2^	7.3 (6.3-8.4)	7.3 (5.2-9.4)	0.500

^1^ Chi-squared test

^2^ 95 % confidence intervals are given in parentheses

Significant changes (p ≤ 0.03) in prevalence at birth were observed for congenital malformations of the circulatory system, the musculoskeletal system (including deformations), the urinary system and the group of other congenital malformations. A substantial increase was evident only for BD of the urinary system (Table 2).

**Table 2:** Malformation prevalence at birth, stratified by anomaly groups before and after the establishment of prenatal screening, per 1000 newborns

Group of birth defect (ICD-10 codes)	1973-2000		2001-2011		p-value^1^
	Number (N) of newborns: 23 822		Number (N) of newborns: 6626		
	N of BD	Prevalence^2^	N of BD	Prevalence^2^	
Congenital malformations of the nervous system (Q00-Q07)	40	1.7 (1.2-2.2)	14	2.1 (1.0-3.2)	0.450
Congenital malformations of eye, ear, face and neck (Q10-Q18)	11	0.5 (0.2-0.7)	6	0.9 (0.2-1.6)	0.174
Congenital malformations of the circulatory system (Q20-Q28)	58	2.4 (1.8-3.1)	7	1.1 (0.3-1.8)	0.032
Congenital malformations of the respiratory system(Q30-Q34)	26	1.1 (0.7-1.5)	3	0.5 (0–1.0)	0.138
Cleft lip and cleft palate (Q35-Q37)	31	1.3 (0.8-1.8)	7	1.1 (0.3-1.8)	0.624
Congenital malformations of the digestive system (Q38-Q45)	29	1.2 (0.8-1.7)	7	1.1 (0.3-1.8)	0.743
Congenital malformations of genital organs (Q50-Q56)	86	3.6 (2.8-4.4)	26	3.9 (2.4-5.4)	0.696
Congenital malformations of the urinary system (Q60-Q64)	21	0.9 (0.5-1.3)	113	17.1 (14.0-20.2)	<0.0001
Congenital malformations and deformations of the musculoskeletal system (Q65-Q79)	323	13.5 (12.1-15.0)	63	9.5 (7.2-11.9)	0.010
Other congenital malformations, excluding multiple (Q80-Q89, excluding Q 89.7)	98	4.1 (3.3-4.9)	12	1.8 (0.8-2.8)	0.007
Chromosomal abnormalities (Q90-Q99)	17	0.7 (0.4-1.1)	5	0.8 (0.1-1.4)	0.807
Multiple congenital malformation, not classified	76	3.2 (2.5-3.9)	20	3.0 (1.7-4.4)	0.937

^1^ p-value for chi-squared test

^2^ 95 % confidence intervals are presented in parentheses

### Decreasing of perinatal mortality

During the 1973–2000 study period, there were 572 cases of perinatal deaths, of which 297 were stillborn. Of these, 506 perinatal deaths (Table 2) and 244 fetal deaths were registered during 1973–2000. Consequently, the perinatal mortality rate decreased from 21.2 per 1000 newborns (95 % CI: 19.4-23.1) in 1973–2000 to 10.0 (95 % CI: 7.6-12.3) in 2001–2011. In the 1990–2000 transitional period, the perinatal mortality rate was 18.4 (95 % CI: 15.2-21.6) per 1000 newborns. Compared to the early and transitional periods, the perinatal mortality for all newborns and newborns without BD decreased two-fold during 2001–2011. Furthermore and relative to the screening period, the perinatal mortality among newborns with any kind of malformation was 5 times higher for the pre-screening period and 2.5-fold for the transition time frame (see Table 3).

**Table 3:** Actual number (N) and prevalence of perinatal deaths (per 1000 newborns) before and after the establishment of prenatal screening

Group of newborns	1973-2000		1990-2000		2001-2011		p-value^1^
	N	Prevalence^2^	N	Prevalence^2^	N	Prevalence^2^	
Newborns with BD (*N* = 1099)	87	106.6 (84.3-129.1)	18	52.9 (32.5-85.9)	6	21.2 (4.3-38.1)	<0.0001^3^
							0.0160^4^
Newborns without BD (*N* = 29 349)	419	18.2 (16.5-19.9)	105	16.5 (13.3-19.6)	60	9.5 (7.1-11.8)	<0.0001^3^
							<0.0001^4^

^1^ p-value is for chi-squared test

^2^ 95 % confidence intervals are presented in parentheses

^3^ For the 1973–2000 and 2001–2011 groups

^4^ For the 1990–2000 and 2001–2011 groups

The logistic regression analysis indicated that mothers who had undergone at least one ultrasound examination during pregnancy (*n* = 9 883), had a lower risk of having a newborn die during the perinatal period [adjusted OR = 0.49 (95 % CI: 0.27-0.89)].

### Prenatal detection rates and terminations of pregnancies due to fetal anomalies

Of 4 596 newborns in the 2000–2007 cohort whose mothers had undergone at least one ultrasound procedure during pregnancy, 182 had a registered BD; of these, 56 were recognized before birth. In addition, 25 pregnancies had been terminated before gestational age of 28 weeks, and had not been reported in the registries. Consequently, the total number of prenatally diagnosed BD were 81 of 232, with an overall prenatal detection rate of 34.9 % (for the complete list stratified by birth defect group see Table 4).

**Table 4:** Prenatal detection rates, stratified by birth defects groups in Monchegorsk in 2000–2007 (including terminations of pregnancies due to fetal anomaly)

Group of birth defect	Number of BD	Number of BD diagnosed before birth	Prenatal detection rate, %
Congenital malformations of the nervous system	25	17	68.0
Congenital malformations of eye, ear, face and neck	3	0	0
Congenital malformations of the circulatory system	12	7	58.3
Congenital malformations of the respiratory system	3	0	0
Cleft lip and cleft palate	3	2	66.7
Other congenital malformations of the digestive system	4	0	0
Congenital malformations of genital organs	21	0	0
Congenital malformations of the urinary system	76	32	42.1
Congenital malformations and deformations of the musculoskeletal system	47	5	10.6
Other congenital malformations, excluding multiple	11	9	81.8
Chromosomal abnormalities	4	1	25.0
Multiple congenital malformation, not classified	23	8	34.8
All birth defects	232	81	34.9

Prenatal diagnoses of the BD pertaining to the 25 pregnancy terminations involved the nervous system (40 %; *n* = 10), the circulatory system (20 %; *n* = 5), multiple malformations (20 %; *n* = 5), congenital malformations of the kidney (8 %; *n* = 2), diaphragm hernia (8 %; *n* = 2), other malformations (4 %; *n* =1).

After inclusion of TOPFA in the mortality analysis, the stillborn rate in 2000–2007 increased to 13.8/1000 (95 % CI = 10.9-13.6) from that based on the registry data [8.5/1000 (95 % CI 5.8-11.1)].The perinatal mortality was 17.7/1000 (95 % I = 14.7-22.0) *versus* 12.4/1000 (95 % CI = 9.2-15.6). Consequently, the estimated reduction in the stillborn rate linked to the abortion of fetuses with severe BD was 38 %, and 30 % for perinatal mortality.

## Discussion

### Perinatal mortality

Our study showed clearly that the implementation of PS had an impact on perinatal mortality and BD prevalence. The five-fold decrease observed in perinatal mortality among newborns after the implementation of PS exceeded that reported for the mentioned Helsinki trial [3]. The latter study covered a 19-month period, while the current work embraced 38-years during which medical practices, technics and management protocols changed drastically. By comparison, the perinatal mortality among newborn without birth defects only halved. The most probable reason is the termination of pregnancies due to incompatible-with-life defects, of which there were 25 in 2000–2007.

The prevalence of TOPFA in 2000–2007 was 5.4 per 1000 births. In Europe, such indicator varies from zero in Ireland, Malta and Poland (where termination of pregnancies is illegal) to 10/1000 births in France [18]. In Russia, the legislation concerning abortions for medical reasons may be considered to be rather liberal as mentioned above [10]. There is no list of BD that are considered as lethal or incompatible with life [10]. All cases of prenatal diagnosis of BD are scrutinized individually. Nevertheless, abortions based on medical indications appear to constitute a small proportion (less than 3 % in 2010). Whereas the reported rate of all pregnancy terminations decreased 49.6 % over the past 20 years in Russia [19], those in Murmansk County decreased from 166 per 100 births in 2000 to 58 per 100 births in 2010 [20]. The proportion of abortions for medical reasons increased from 2.2 % in 2000 to 4.2 % in 2006, with a subsequent decrease to 2.4 % in 2008. The decline in overall terminations rate could be due to the improving socio-economic situation, the lowering in social indicators for abortions in both 2003 and 2011 [21, 22] from 13 to one (pregnancy resulting from sexual assault), and a cutback in the number of medical criteria in 2007 [10]. It is important to note that these changes do not apply to BD.

At first glance, one might suggest that the establishment of screening has not influenced pregnancy continuation. It could be argued that due to the multiplicity of medical reasons for termination the dynamics of abortion incidence could not truly represent the influence of PS. However, our results contradict this. Adjustment by year in the logistic regression analysis excluded a potential influence of medical progress over time, and this suggested that the effect of decreasing the number of perinatal deaths was related to the early detection of pathological conditions and medical intervention. More accurate determination of gestational age was likely an issue as well.

### Birth prevalence of birth defects

The observed increase in the birth prevalence of BD during 2001–2011 (Table 2) may largely attributed to an increase in the prevalence of the urinary system malformations. It is correlated with a high prenatal detection rate for this malformation group and may indicate over-diagnosis. This appears to be so for Q62.0 (congenital hydronephrosis) and Q63.0 (another malformations of kidney, unspecified), as they represent more than a half of all urinary malformations in Monchegorsk. Similarly, and based on prenatal ultrasound examination, Luck [23] also reports a high prevalence of hydronephrosis (95 cases from 140 cases of all BD). Over-diagnosis of this malformation could be due to difficulties in differentiating between the physiological size of renal pelvis and renal pathological dilatation, although national guidelines at the time the PS was conducted were absent. The prevalence of cardiovascular BD has significantly changed after PS implementation. This group consists of rather severe defects, which often are incompatible with life and thus their prenatal diagnosis can lead to pregnancy termination. Consequently, the observed decrease in their prevalence might reflect improved diagnoses before birth with subsequent terminations (20 % of total). We did not find an increase in the prevalence of ventricle and atrial septal defects as reported by other authors [24, 25]. Perhaps our short follow-up period, which ended with the discharge from the maternity ward, may have precluded it.

The prevalence of the most severe BD that require mandatory registration was stable over the two main study periods. It constitutes a heterogeneous group of defects, some of which could be corrected by surgery, while others are incompatible with life. Perhaps an increase in the prevalence of the former may have increased pregnancy termination since after the establishment of PS no cases were registered at birth of anencephaly, diaphragmatic hernia and omphalocele; and only one case each of cleft palate and gastrosсhisis. The prevalence of Down syndrome decreased, but not statistically so (comparison was limited by relatively small n, namely 6606 newborns after 2000).

A decrease in musculoskeletal malformation’s prevalence (see Table 2) did not correlate with their detection rate, which is one of the lowest (see Table 4). Neither does the observed absence of pregnancy terminations due to these malformations seem likely. Presumably, a true prevalence decrease occurred. Possibly the mothers were transferred for delivery to central clinics (Murmansk, Saint-Petersburg, Moscow) and, accordingly, information about such newborns were not included in the registries.

### Prenatal detection rates and perinatal prevalence

The observed antenatal detection rate of malformations was low – less than a half of those diagnosed after birth. It was lower than that disclosed in the EUROSCAN study [1, 5]. However, the EUROSCAN investigation considered detection rates for only 11 severe defects, while our study considered all BD. Taking into account all malformations, the prenatal detection rate in Europe was 31.8 % in 2008–2012 [26] and thus was comparable to ours. Nevertheless, the prenatal detection rate in our study could be an overestimate for some groups of minor defects that subsequently could not be recognized during the first week of life. Thus, our estimation is most valid for easily diagnosed defects, such as of the nervous system, orofacial or muscular-skeletal anomalies; for these, the EUROCAT detection rates were higher [26].

Our data is similar to the Russian studies mentioned earlier [11], with urinary malformations and cardiovascular defects the exceptions. The observed antenatal prevalence of 1.2 % is comparable to that reported in a systematic review covering the late 1990s (based primarily on USA and European studies) [27].

The Russian system of perinatal diagnostics has primarily focused on municipal out-patients departments (“women’s consultations”), staffed with specialists in ultrasound diagnostics. The mean number of examinations per woman during pregnancy was high, although of rather poor quality, as half of the malformations were not diagnosed before birth [28]. Some Russian researchers have shown that the municipal administration of PS has been inefficient, and suggested that regional centers with more experienced staff be established [29].

A primary feature of the PS program in Monchegorsk is the poor detection of chromosomal defects. For example, no diagnosis of Down syndrome was made before birth. The likely reason is that Monchegorsk is a relatively small city (about 45 000 inhabitants compared to around 300 000 in Murmansk), and such municipal hospitals have been recognized to have problems with both the quality of the medical equipment and of medical staff training, especially during the first years of PS. Chromosomal defects are difficult recognize and diagnose. They were more frequently addressed in regional clinics, which are staffed with genetic specialists and consultants for the second stage of PS, as described above.

### Strengths and limitations

Our study is the first in Northern Russia to analyze the effectiveness of PS at the population level that is based on validated sources of information, including TOPFA data. Furthermore, the two birth registries used provided full coverage of all pregnancy’s outcomes, although our study was limited to information about BD diagnosed during the first week of life. The latter could have led to a possible underestimation of the prevalence of small birth defects, although it did not influence the prenatal mortality. We did not have data on TOPFA for the whole study period, and consequently could not include them in the prevalence analyses. Thus, we aimed to estimate prevalence at birth and, we suppose, that inclusion of TOPFA would have increased the estimated prevalence somewhat. In this instance, prevalence of severe BD would also have increased. Some pregnant women suspected to give birth to newborns with serious BD did so at major hospitals in Murmansk, Moscow or Saint Petersburg and their neonates were not included in our analysis. Inclusion of such newborns could increase the prenatal detection rates, but the proportion of such cases was not high. For example, according to the data provided by the Monchegorsk Hospital six newborns (6.8 %) with prenatally diagnosed BD (2.5 % of all newborns with BD) were born in Murmansk or Moscow. Due to differences in coding between registries and the large proportion (up to 5 %) of missing information, our regression model did not include all possible confounders such as comorbidities of mothers and complications of pregnancy, previous history of stillborn and maternal socio-economic status.

## Conclusion

The birth prevalence of BD doubled after the introduction of routine PS, but less than half of the defects were diagnosed before birth. Improved detection of severe malformations with subsequent termination is likely to have been the main contributor to the observed decrease in perinatal mortality during 2001–2011 in Murmansk County, Russia.

## Abbreviations

BD: birth defect
EUROCAT: European Surveillance of Congenital Anomalies
ICD-10: the International statistical classification of diseases and related health problems, tenth revision
KBR: Kola Birth Registry
MCBR: Murmansk County Birth Registry
MCHA: Murmansk County Health Authority
PS: prenatal screening
TOPFA: termination of pregnancy due to fetal anomaly
